# Cellular and complement-dependent cytotoxicity of Ep-CAM-specific monoclonal antibody MT201 against breast cancer cell lines

**DOI:** 10.1038/sj.bjc.6602310

**Published:** 2005-01-11

**Authors:** N Prang, S Preithner, K Brischwein, P Göster, A Wöppel, J Müller, C Steiger, M Peters, P A Baeuerle, A J da Silva

**Affiliations:** 1 Micromet AG, Staffelseestrasse 2, Munich 81477, Germany

**Keywords:** MT201, breast cancer, ADCC, CDC, Ep-CAM, trastuzumab

## Abstract

MT201 is a fully human monoclonal IgG1 antibody with moderate affinity for epithelial cell adhesion molecule (Ep-CAM) being clinically developed for the treatment of carcinomas. Like many other clinically validated IgG1 monoclonal antibodies, MT201 primarily acts by antibody-dependent cellular cytotoxicity (ADCC) and complement-dependent cytotoxicity (CDC). Here, we analysed ADCC and CDC induced by MT201 and, as reference, trastuzumab against a panel of nine human breast cancer cell lines expressing distinct surface levels of Ep-CAM and human epithelial growth factor receptor type 2 antigen. Maximal cell lysis by ADCC by MT201 and trastuzumab in the presence of peripheral mononuclear cells did not significantly differ when averaged over the nine cell lines, but showed marked differences with respect to individual cell lines. The extent of cell lysis at intermediate surface target density was highly variable, suggesting a dominant influence of other susceptibility factors. Only one breast cancer cell line was eliminated via CDC, but only by MT201. Resistance to CDC appeared to correlate with high expression levels of complement resistance factors. Our present data as well as recent data on the prevalence and prognostic relevance of Ep-CAM expression in metastatic breast cancer suggest that Ep-CAM-specific monoclonal IgG1 antibodies may have a significant therapeutic potential in the treatment of breast cancer.

Over the past few decades, a considerable effort has been devoted to the discovery and development of novel chemotherapeutics for the treatment of breast cancer (Burstein *et al*, 2001; Cristofanilli and Hortobagyi, 2001). However, although the initial responses to chemotherapeutic regimens are frequently positive, their duration is often brief and the majority of patients die shortly after relapsing. In the US alone, on average approximately 40 000 women die from the disease every year (Winer *et al*, 2001; Weir *et al*, 2003). Commonly, the cause for chemotherapy failure is the emergence and subsequent expansion of drug-resistant tumour cells. This shortfall in long-term efficacy has led to the quest for novel forms of therapy that act by a different mode of action. The last decade has seen the successful development of novel immunoglobulin (Ig)-based therapeutics, either in the form of native monoclonal antibodies (mAbs) or as conjugates with radionuclides and toxins (Clynes *et al*, 2000; Carter, 2001; White *et al*, 2001; Wels *et al*, 2003; Wiercioch *et al*, 2003). Unlike chemotherapeutics, Ig-based therapies can be designed to target specifically tumour cells via the recognition of antigens overexpressed on their surface. Mechanisms of mAb-based therapies can range from antiproliferation and apoptosis by the blockade of receptor/ligand interactions, the targeted delivery of prodrugs, toxins, radioisotopes and chemotherapeutics to the engagement of immune effector mechanisms such as complement-dependent cytotoxicity (CDC) and antigen-dependent cellular cytotoxicity (ADCC). Recently, several mAbs that predominantly act by ADCC and CDC have been approved for the treatment of cancer patients. These include chimaeric IgG1 mAb rituximab (Rituxan®) binding to the B-cell differentiation antigen CD20 for the treatment of B-cell lymphomas (Grillo-Lopez *et al*, 1999; Smith, 2003), humanised IgG1 mAb trastuzumab (Herceptin®) targeting HER-2 (human epithelial growth factor receptor type 2) overexpressed in a subgroup of breast cancers (Vogel *et al*, 2001), humanised IgG1 alemtuzumab (Campath®) targeting the differentiation antigen CD52 for the treatment of B-cell chronic lymphocytic leukaemia (Hale *et al*, 1998; Kottaridis *et al*, 2000; Faulkner *et al*, 2004) and edrecolomab (Panorex®), a murine IgG2a mAb targeting Ep-CAM (epithelial cell adhesion molecule), which gained temporary approval in Germany for the treatment of colorectal carcinoma (Riethmuller *et al*, 1994; Gruber *et al*, 1996; Schwartzberg, 2001; White *et al*, 2001). Several other mAbs are currently at advanced stages of clinical development.

Epithelial cell adhesion molecule (also referred to as 17-1A, KSA, GA733-2 or ESA) is a 40 kDa transmembrane glycoprotein that is expressed at relatively low levels on basolateral cell surfaces of most human simple epithelia (Balzar *et al*, 1999). Expression of Ep-CAM is highest during embryogenesis and in association with metaplastic or neoplastic changes, and lowest in mature epithelial tissues. Ep-CAM overexpressing cells tend to segregate from normal cells correlating with the development of a proliferative and malignant phenotype (Winter *et al*, 2003). Epithelial cell adhesion molecule is overexpressed on the majority of human carcinomas, and frequently expressed *de novo* on squamous cell carcinoma of head and neck, bladder and lung (Litvinov *et al*, 1996; Balzar *et al*, 1999; Went *et al*, 2004). For example, a very recent study determined by quantitative polymerase chain reaction a >400-fold overexpression of Ep-CAM in ovarian cancer tissue relative to normal ovarian epithelium (Kim *et al*, 2003).

Another antibody target overexpressed on various carcinomas is the HER-2 (also called ErB2), a 185 kDa transmembrane cell surface glycoprotein and member of the protein tyrosine kinase receptor family (Schechter *et al*, 1984; Yamamoto *et al*, 1986). Although no ligand for HER-2 was found to date, the molecule has been shown to heterodimerise with other HER family members, contributing to the comodulation of their activity in the regulation of cell growth, differentiation and survival. Overexpression of HER-2 is thought to play a role in the aberrant proliferation of cancer cells (Kallioniemi *et al*, 1991; Rilke *et al*, 1991).

In a retrospective study of 205 lymph node-positive breast cancer patients, Gastl and co-workers (Spizzo *et al*, 2002) compared the prognostic impact of HER-2 and Ep-CAM overexpression on patient survival. A highly significant negative correlation was found between the expression level of Ep-CAM and disease-free and overall survival. Even patients expressing Ep-CAM at lower level and not on every tumour cell showed a survival disadvantage compared to patients with Ep-CAM-negative tumours. High-level Ep-CAM overexpression was detected with 35.6% of patients, while expression of HER-2 was found elevated in 19.5% of the patients, with overexpression of both targets in 13.2%. Hence, approximately 20% of breast cancer patients overexpressed Ep-CAM but not HER-2. Only 10% of breast cancer patients had Ep-CAM-negative tumours. These studies indicated that therapies targeting HER-2 or Ep-CAM overexpressing tumours, which may be more aggressive and evasive, are likely to exert a significant impact on overall survival of breast cancer patients.

The monoclonal IgG1 antibody trastuzumab targeting HER-2 has been approved as monotherapy and in combination with docetaxel (Taxotere®) for the treatment of patients with metastatic breast cancer (Dieras *et al*, 2001; Leyland-Jones, 2001; Slamon and Pegram, 2001). Trastuzumab is believed to mediate killing of HER-2 overexpressing tumour cells via ADCC, CDC as well as interference with HER-2 signalling (Sliwkowski *et al*, 1999). However, as only approximately 15% of breast cancer patients highly overexpress HER-2, and thus benefit from trastuzumab treatment (Spizzo *et al*, 2002), the majority of breast cancer patients are still in need of IgG1 antibody-based therapies targeting other more widespread antigens.

MT201 is a fully human IgG1 mAb that binds Ep-CAM with moderate affinity (Naundorf *et al*, 2002). A moderate affinity was chosen in view of toxicity issues with two high-affinity anti-Ep-CAM mAbs (Khor *et al*, 1997; LoBuglio *et al*, 1997; Saleh *et al*, 1997; de Bono *et al*, 2002), and an otherwise very benign safety profile of the moderate affinity murine IgG2a mAb edrecolomab (Panorex®) (Braun *et al*, 1999; Kirchner *et al*, 2002; Punt *et al*, 2002). Preclinical studies have shown that MT201 mediates target cell lysis via ADCC and CDC in various human cancer cell lines (Naundorf *et al*, 2002). Efficacy was also shown *in vivo* in nude mouse xenograft models using the Ep-CAM-positive human colon cancer cell line HT-29 (Naundorf *et al*, 2002), and *ex vivo* using primary human ovarian tumour samples (Xiang *et al*, 2003).

Here, we report on *in vitro* studies evaluating the ADCC- and CDC-mediated cytotoxic efficacy of MT201 against a panel of nine human breast carcinoma cell lines. In all experiments, efficacy of MT201 was compared to that of trastuzumab as a reference. Surface expression of both Ep-CAM and HER-2 was determined for all cell lines, and mAb-mediated cytotoxicity analysed in relationship to target density and complement resistance factor expression. At concentrations corresponding to targeted serum trough levels, MT201 appeared equally active in ADCC as trastuzumab, suggesting that clinical administration of MT201 could also provide benefit to breast cancer patients. Given the more frequent overexpression of Ep-CAM on metastatic breast cancer than HER-2, MT201 may have a comparable if not higher therapeutic potential than HER-2-specific antibodies.

## MATERIALS AND METHODS

### Cell lines and reagents

The KATO III human gastric carcinoma cell line was obtained from the European Collection of Cell Cultures (ECACC, Salisbury, UK). The various breast cancer cell lines were obtained from either the German Collection of Microorganisms and Cell Cultures (DSMZ, Braunschweig, Germany) or from the American Type Culture Collections (ATCC, Manassas, VA, USA). Cells were cultured in RPMI media (Invitrogen, Karlsruhe, Germany), supplemented with 10% foetal bovine serum (Invitrogen, Karlsruhe, Germany), at 37°C, in a 5% CO_2_ chamber. Human sera for CDC assays were obtained from healthy donors and immediately stored at −20°C after centrifugation of coagulated peripheral blood. The variable domains of MT201 were isolated from a human IgD-positive B-cell repertoire by guided selection and phage display and combined with human IgG1 constant domains as described previously (Raum *et al*, 2001). MT201 was produced by Chinese hamster ovary (CHO) cells and purified to homogeneity by standard procedures. Trastuzumab (anti-HER-2, Herceptin®) mAb was obtained from Hoffmann La-Roche (Grenzach-Whylen, Germany). The mouse anti-Ep-CAM IgG2a mAb M79 was a kind gift from Drs Peter Kufer and Judy Johnson (Institute for Immunology, Ludwig-Maximilians University Munich, Germany) (Gottlinger *et al*, 1986). The mouse anti-HER-2 IgG1 mAb 9G6 was obtained from BD Biosciences (Heidelberg, Germany). The murine IgG1 anti-CD55 mAb was purchased from the International Blood Group Reference Laboratory (IBGRL, Bristol, UK) and the murine IgG2b anti-CD59 mAb from Pharmingen (Heidelberg, Germany). FITC-conjugated anti-mouse IgG was purchased from DAKO (Hamburg, Germany), while FITC-conjugated anti-human IgG was purchased from ICN (Eschwege, Germany).

### Saturation binding assay

Saturation binding assays were carried out using the QIFIKIT from DAKO following the manufacturer's recommendations (Hamburg, Germany). All steps were performed on ice. Briefly, cells grown under regular growth conditions were trypsinised for 5 min, sedimented by centrifugation at room temperature for 5–7 min at 200–450 × **g** and resuspended to 10^6^ cells ml^−1^. A measure of 50 *μ*l of cells (i.e. 5 × 10^4^) are then added to each well of a 96-well plate, in duplicate for each experimental condition. After centrifugation of the culture plates at 350 × **g** for 4 min, cells were resuspended in 50 *μ*l of a three-fold serial dilution of the primary antibody in FACS (fluorescence-activated cell sorting) buffer as follows: 300, 100, 33.3, 11.1, 3.7, 1.2, 0.4 and 0.13 *μ*g ml^−1^ and a blank (FACS buffer alone), and then incubated at 2–8°C for 45 min. To quantify target expression, 100 *μ*l of either set-up beads or calibration beads are pipetted into two separate wells. Cells and the beads were then sedimented by centrifugation at 300 × **g** at 4°C for 4 min and washed twice in 200 *μ*l of FACS buffer. Cells and calibration beads are then resuspended in 100 *μ*l of the appropriate secondary antibody (anti-human IgG FITC or anti-mouse IgG FITC). Following incubation at 2–8°C for 45 min, samples are washed three times as above and resuspended in 200 *μ*l of FACS buffer. Staining is then analysed by flow cytometry using a FacsCalibur (BD Biosciences, Heidelberg, Germany) and recording 2500 events. Determination of surface antigen expression is then determined by extrapolation from curves derived from the calibration beads. Dose–response curves were computed by nonlinear regression analysis using a four-parameter fit model using Prism software (GraphPad Software, San Diego, CA, USA).

### ADCC assays

Target cells grown under regular culture conditions were trypsinised for 5 min, sedimented by centrifugation and resuspended in culture medium to a concentration of 10^6^ cells ml^−1^. Cells (5 × 10^6^) are then mixed with calcein to a final concentration of 0.2 *μ*M and incubated at 37°C for 25 min. After one wash with PBS, cells were resuspended in culture media and adjusted to a concentration of 3 × 10^5^ cells ml^−1^. Separately, peripheral blood mononuclear cells (PBMC) were prepared following conventional procedures (enriched by Ficoll–Hypaque gradient centrifugation), washed and resuspended at 6 × 10^6^ ml^−1^. Equal volumes of target and effector cell suspensions are mixed resulting in a final ratio of effector to target cells (E : T) of 20 : 1, and 100 *μ*l of this cell mixture is then added per well of a 96-well plate. This is followed by the addition of 20 *μ*l of an antibody solution, previously diluted in a 1 : 4 series, resulting in a final concentration ranging from 0.2 to 50 000 ng ml^−1^. Cells are then incubated for 3.5 h at 37°C, after which 50 *μ*l of propidium iodide (PI) solution is added to yield a final concentration of 1 *μ*g ml^−1^. The cells are further incubated for another 30 min at 37°C and 20 000 cells are then analysed by Flow Cytometry using a FacsCalibur (BD Biosciences, Heidelberg, Germany). Dose–response curves were computed by nonlinear regression analysis using a four-parameter fit model using Prism software (GraphPad Software, San Diego, CA, USA). All experiments were performed in triplicate.

### CDC assays

Target cells grown under regular culture conditions were trypsinised for 5 min and resuspended in RPMI media at a concentration of 5 × 10^6^ cells per 1.7 ml of media. Calcein was added to a final concentration of 10 *μ*M and the cells incubated for 30 min at 37°C. Cells were then washed twice in PBS and resuspended in prewarmed RPMI media (37°C) to a final concentration of 0.625 × 10^5^ cells ml^−1^. To each 160 *μ*l of cell suspension, 20 *μ*l of cold human serum and 20 *μ*l of a 1 : 2 serial dilution of MT201 were added. In all, 10 concentrations of MT201 ranging from 95 to 50 000 ng ml^−1^ final, plus a blank control, were used. After incubation of the cell cultures for 45 min at 25°C, cells were sedimented and 100 *μ*l of the supernatant analysed by fluorometry to measure cell death (calcein release) at 485/535 nm using SPECTRAFluor Plus reader (BD Biosciences, Heidelberg, Germany). Total lysis of the cells was achieved by solubilising a non-MT201-treated control sample with 20 *μ*l of a 9% solution of Triton X-100. Specific lysis was then determined as the percentage of the measured calcein release from MT201-treated and the Triton X-100-solubilised sample. Data analyses were carried out using Excel (Microsoft, Munich, Germany) and Prism software (GraphPad Software, San Diego, CA, USA). All experiments were performed in triplicate.

## RESULTS

### Ep-CAM and HER-2 surface expression on nine breast cancer cell lines

We quantitated the surface target density of Ep-CAM and HER-2 on nine breast cancer cell lines, which were of different tumour source and differed with respect to the status of oestrogen receptor expression, HER-2 amplification and expression of FGF and TGF*α* (Table 1 ). Saturation binding assays were performed under conditions that prevent downmodulation of antigens by antibody binding. The technique used microbeads coated with predetermined concentrations of antibody as calibration standards and required murine antibodies. Murine anti-Ep-CAM mAb M79 (Gottlinger *et al*, 1986) and anti-HER-2 mAb 9G6 (van de Vijver *et al*, 1988) were selected because they had binding affinities similar to MT201 and trastuzumab, respectively. A 5-min trypsin treatment used for detachment of cells from culture dishes did not result in loss of surface antigen density when compared to EDTA treatment (data not shown).

The Ep-CAM surface density on the nine breast cancer cell lines ranged from 6.71 × 10^5^ binding sites/cell on MT-3 cells to just 1.7 × 10^3^ on MDA-MB-231 cells (Table 2 ). Analysis of human Ep-CAM-negative CHO cells revealed background binding in the order of 10^3^ sites/cell (data not shown), indicating that the MDA-MB-231 cell line can be considered as Ep-CAM negative. Except for the latter, the remaining eight cell lines all expressed more than 10^5^ sites/cell, and in three of the cell lines (MT-3, ZR-751 and MCF7), expression levels exceeded 2 × 10^5^ molecules. HER-2 expression also varied over a broad range from 9.76 × 10^5^ on SKBR3 to just 1.4 × 10^4^ on MDA-MB-231 cells. However, the relative expression levels of HER-2 were not as evenly distributed as for Ep-CAM. Although two cell lines expressed HER-2 above 2 × 10^5^ (the other cell line being BT474 at 6.91 × 10^5^), only one other cell line expressed HER-2 above 10^5^ (MDA-MD-453 at 1.61 × 10^5^), while all other cell lines expressed less than 6.9 × 10^4^ molecules per cells (Table 2). The median expression for Ep-CAM on the nine breast cancer cell lines was 1.49 × 10^5^, and for HER-2 was 3.16 × 10^4^, showing a 4.4-fold difference. Ep-CAM and HER-2 expression on the gastric carcinoma KATO III reference cell line reached 8.93 × 10^5^ and 2.3 × 10^4^ binding sites/cell, respectively.

### ADCC by MT201 and trastuzumab against nine breast cancer cell lines

**Figure 1:**
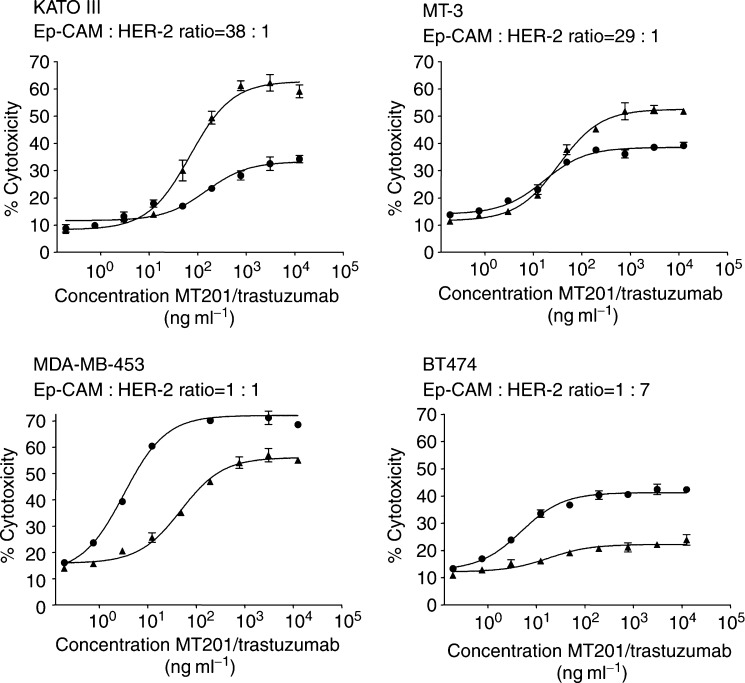
Examples of ADCC dose–response analyses with MT201 and trastuzumab. Three human breast cancer cell lines expressing different relative levels of Ep-CAM and HER-2 were selected. The KATO III cell line served as reference. Increasing concentrations of MT201 (triangles) or trastuzumab (circles) were tested with human PBMC as effector cells at an E : T ratio of 20 : 1 for 4 h. Specific cytotoxicity refers to the difference in cell lysis seen between antibody-treated and control cell cultures. Cell lysis was determined by staining target cells with PI and subsequent analysis by FACS.

The effector cell-mediated cytotoxic efficacy of MT201 and trastuzumab was analysed using the panel of nine different human breast carcinoma cell lines of various antibody target antigen densities (Tables 1 and 2). The human gastric carcinoma cell line KATO III, which has previously been shown to express high levels of Ep-CAM and to be sensitive to MT201-mediated ADCC and CDC (Naundorf *et al*, 2002), served as reference cell line. As source of effector cells for ADCC, PBMC from healthy donors were used at an E : T ratio of 20 : 1. Analysis of enriched effector cell subpopulations showed that natural killer cells typically contributed >90% of ADCC activity within PBMC (Prang *et al*, submitted). The same donor PBMCs were used for direct comparison of MT201 and trastuzumab with each cell line tested. Dose–response analyses allowed determining EC_50_ values as well as the percentage of maximal cell lysis after a 4-h ADCC reaction (Figure 1). The proportion of alive and lysed cells was determined by FACS analysis using the dye PI (Naundorf *et al*, 2002). An isotype control IgG1 failed to induce target cell lysis above background (data not shown). Figure 1 shows representative examples of ADCC dose–response curves derived from four cell lines expressing different relative levels of Ep-CAM and HER-2 (see Table 2).

With the cell lines KATO III and MT-3 cells, where Ep-CAM is more highly expressed than HER-2, MT201 reached higher levels of cell lysis in the 4-h assay than trastuzumab. With MDA-MB-453 cells, which expressed comparable levels of Ep-CAM and HER-2, cells were more susceptible to lysis by trastuzumab than MT201. Finally, with BT474 cells, which expressed approximately seven times more HER-2 than Ep-CAM, trastuzumab was as active as on MDA-MB-453 cells, while MT201 showed very little activity. ADCC values for all cell lines expressed as percent maximal-specific cell lysis are summarized in Table 3 . The range of specific lysis induced by MT201 varied between a maximum of 44.3% for MDA-MB-453 to a low of 6.2% for the MDA-MB-231 line. These levels were generally comparable to those achieved by trastuzumab, where the range varied from a maximum of 64.1% for MDA-MB-453 cells to 7.6% for BT20 cells. The median lysis over nine breast cancer cell lines was 20.2±14.6% for MT201, and 27.9±15.0% for trastuzumab, and not significantly different. Trastuzumab was generally able to yield higher cell lysis at a lower antigen expression level than MT201 as reflected by lower EC_50_ values (Table 4 ). For MT201, EC_50_ values varied from 461.4 ng ml^−1^ for SKBR3 cells to 11.9 ng ml^−1^ for BT474. For Herceptin, they ranged from 138 ng ml^−1^ for BT20 cells to 4.4 ng ml^−1^ for MDA-MB-453 cells. BT20 cells yielded high EC_50_ values for the two antibodies, which also correlated with the lowest specific maximal lysis observed (compare Tables 3 and 4).

**Figure 2:**
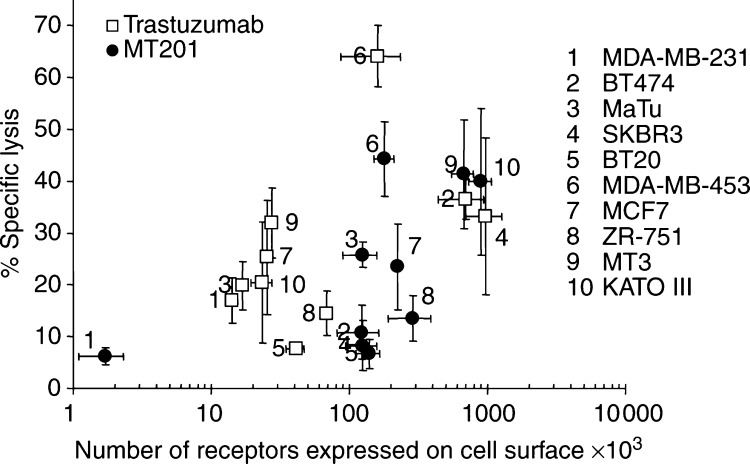
Correlation between target densities of Ep-CAM and HER-2 with ADCC of MT201 and trastuzumab. Expression of Ep-CAM and HER-2 on nine breast cancer cell lines was determined by saturation binding assays as shown in Table 2. ADCC refers to maximal specific cell lysis as shown in Table 3.

Overall, the specific cytotoxicity of MT201 and trastuzumab correlated only to a low degree with the respective target densities on each cell line (Figure 2). With four cell lines of high target density for Ep-CAM and HER-2, maximal cell lysis by MT201 and trastuzumab was very similar.

### CDC by MT201 and trastuzumab on nine breast cancer cell lines

We next assessed whether MT201 and trastuzumab could induce breast cancer cell lysis via CDC. Freshly purified human serum from healthy volunteers was used as source of complement. The only two cell lines susceptible to CDC by MT201 were MT-3 cells and the KATO III reference cell line (Table 5 ). No MT-3 and KATO III target cell lysis was observed when cells were incubated with an isotype control antibody, or after heat treatment of the human serum (data not shown). None of the 10 cell lines tested showed any CDC by trastuzumab, suggesting that neither CDC nor direct receptor-mediated mechanisms could substantially contribute to cell lysis under our experimental conditions.

The response of the various cell lines to MT201-induced CDC appeared to correlate negatively with the expression of certain complement resistance factors (Table 5). The best correlation to CDC resistance was seen with the expression level of CD59. KATO III cells, which exhibited the greatest sensitivity to CDC, expressed the lowest level of CD59, while they still expressed resistance factors CD55 and CD46 to a level comparable to that of the other cell lines. Of all breast cancer cell lines tested, MT-3 cells was the only cell line susceptible to CDC. It had the lowest level of CD59 and highest level of Ep-CAM expression.

## DISCUSSION

The modes of action of MT201 and trastuzumab should be highly related. Both antibodies recognise targets that are overexpressed on tumour cells relative to normal tissue, and overexpression of both targets was found to correlate negatively with overall patient survival. Both antibodies share the same human Fc*γ*1 (constant region of Ig type 1) portion that, among all human Fc*γ* subtypes, is best suited to mediate ADCC (Trinchieri and Valiante, 1993; Yokoyama and Plougastel, 2003). Differences between MT201 and trastuzumab may therefore largely relate to their target binding affinities and the biology and quality of the recognised antigens.

Ep-CAM has no known signalling functions and to date no antibody against the molecule has been reported to significantly affect cell proliferation or survival. In contrast, antibody binding to HER-2 is thought to induce receptor-mediated intracellular signalling leading to antiproliferative effects (Chazin *et al*, 1992). However, we failed to observe any significant cytotoxic activity by trastuzumab against a panel of nine different breast cell lines in the absence of effector cells (data not shown). While we cannot exclude that incubation times longer than those used in our assays may be needed in order to detect immune effector-independent activities of trastuzumab, the only measurable cytotoxic activity that could be observed for trastuzumab in our study was ADCC. It might be possible that the somewhat higher ADCC activity of trastuzumab compared to MT201 was due to sensitisation of target cells via a receptor-mediated cytotoxic mechanism. Such a sensitisation did, however, not manifest in the presence of complement. A simpler explanation for the lower EC_50_ values of ADCC of trastuzumab compared to MT201 is that the anti-HER-2 antibody has an approximately 100-fold higher binding affinity than MT201, leading to higher surface density of bound antibody. Similarly, higher levels of ADCC were achieved by MT201 over trastuzumab with cell lines expressing high levels of Ep-CAM and low levels of HER-2. Hence, the lower target binding affinity of MT201 may be compensated by a higher expression level and prevalence of the Ep-CAM over the HER-2 target. Moreover, in this study, we have selected ADCC assay conditions suitable to identify fine differences in ADCC activity between trastuzumab and MT201. In a clinical setting, exposure times of tumour cells to effector cells and antibody will not be 4 h but several weeks. As a consequence, such differences may diminish and much higher levels of ADCC might be observed for both antibodies.

An interesting observation was that six breast cancer cell lines differed only by a factor of two in Ep-CAM expression, namely between 1.24 and 2.22 × 10^5^ sites/cell. Despite a very similar target density, these six cell lines drastically differed with respect to ADCC susceptibility, namely between 6.7 and 44.3% specific lysis. This suggests that at an intermediate Ep-CAM target density other factors that can impact on susceptibility to cellular cytotoxic may become dominant. Such factors could include overexpressed protease inhibitors (serpins) or antiapoptotic proteins involved in immune evasion (Trowsdale and Parham, 2004). Likewise, the correlation of ADCC induced by trastuzumab with HER-2 expression levels was not very strong.

The absence of any CDC activity by trastuzumab suggests that the antibody cannot promote assembly of the entire complement cascade. Likewise, it is possible that CDC requires a rather high target antigen density and that a cell line such as SKBR3 with the highest density for HER-2 (similar to that of Ep-CAM on MT-3 cells) also happened to express high levels of complement resistance factor CD59, whereas MT-3 cells with a comparably high Ep-CAM target density happened to express low levels of CD59. Ep-CAM, which forms a tetramer (Balzar *et al*, 2001), may also allow for a closer binding of two IgG1 molecules and can thereby better mediate C1q assembly and CDC than trastuzumab binding to HER-2. The contribution of CDC to the cytotoxic activity of IgG1 in solid tumours may generally be underestimated when studying cultured cell lines. We have here observed a high expression level of complement resistance factors CD46, CD55 and CD59 on almost all cell lines. IHC studies of tumour samples from various stages of colorectal cancer, however, showed that CD59 and CD55 expression is far less frequent than seen with our tumour cell lines (Koretz *et al*, 1993; Hosch *et al*, 2001). As CD59 can interact with CD2 on T cells, there may even be a selective pressure against CD59 expression on tumour cells to avoid interaction with T cells (Hahn *et al*, 1992; Koretz *et al*, 1993).

In designing a novel antibody against Ep-CAM, experiences with other Ep-CAM antibodies and IgG1 mAb therapies need to be considered. Unlike the murine antibody edrecolomab (Panorex®), which rapidly loses efficacy in man due to neutralisation by anti-edrecolomab antibodies, a novel anti-Ep-CAM antibody should be a human IgG1 and of lowest possible immunogenicity. Only these two features can guarantee maximal serum half-life and efficacy through optimal compatibility with human immune effector mechanisms (ADCC and CDC). This goal was achieved with MT201 by isolating Ep-CAM-specific VH and VL domains from a human IgD-positive B-cell repertoire essentially free of somatic mutations (Raum *et al*, 2001), and their fusion to human IgG1 constant domains. Moreover, from a safety standpoint, an anti-Ep-CAM antibody must be able to discriminate between the low levels of Ep-CAM found on normal tissues and the Ep-CAM overexpressed on malignant tissues. There is evidence from clinical trials with high-affinity anti-Ep-CAM antibodies, which caused acute pancreatitis with an increase in serum levels of amylase and lipase (Better *et al*, 2002), that Ep-CAM on normal tissue can be recognised by these antibodies. On the other hand, edrecolomab with a moderate affinity in the range of MT201 (Naundorf *et al*, 2002) showed in more than 3000 treated patients a relatively benign safety profile (Riethmuller *et al*, 1994; Punt *et al*, 2002). An additional therapeutic window for MT201 may come from an engagement and sequestration of Ep-CAM within homotypic cell adhesion zones present between normal epithelial cells. Antibody binding to the few disengaged Ep-CAM molecules on the basolateral side of normal epithelial cells may not suffice to form cytolytic synapse with effector cells nor for effective complement fixation. Moreover, *in vitro* cell culture reaggregation assays have shown that high-affinity anti-Ep-CAM antibodies such as 323/A3 can block cell adhesion at concentrations of 10 *μ*g ml^−1^ (Litvinov *et al*, 1994). Therefore, high-affinity but not intermediate affinity anti-Ep-CAM mAbs may be able to damage directly the insulating function of normal (pancreatic) epithelia by neutralising Ep-CAM's epithelial cell adhesion function.

In light of the previous experience with anti-Ep-CAM antibodies, we selected a moderate Ep-CAM binding affinity for development of MT201. This required to compromise on a reduced ADCC activity at low antibody concentrations. The reduced target affinity of MT201 may, however, be effectively counter-balanced by two factors. One is the high-level overexpression of Ep-CAM on many human carcinomas and the high prevalence of Ep-CAM overexpression. The other is that IgG1 therapies for cancer treatment require relatively high serum trough levels, typically in excess of 10 *μ*g ml^−1^, at which concentration target binding is in saturation even for low-affinity antibodies. High trough levels are counter-intuitive in light of the relatively high target binding affinities of most IgG1 mAb therapies. They may relate to the fact that efficacy is mostly mediated by effector cells bearing the low-affinity Fc*γ* receptor type III (CD16) (Hazenbos *et al*, 1996; Clynes *et al*, 2000). Therefore, it may be largely the affinity of IgG1 for CD16 and not for the target antigen that is rate limiting for efficacy. Furthermore, the quenching of this interaction by extra IgG in serum will require higher *in vivo* mAb concentrations for competition (Naundorf *et al*, 2002). A key role for CD16 in ADCC is supported by the observation that a CD16 polymorphism had a profound impact on the clinical efficacy of rituximab (Cartron *et al*, 2002). This polymorphism affects the affinity of CD16 for IgG1 by a single amino-acid difference.

The therapeutic potential of MT201 and trastuzumab in patients will be affected by several other parameters. Tumour penetration of antibodies was found to be improved with reduced target affinity (Weiner and Thakur, 2001), which could be an advantage for MT201. Likewise, beneficial pharmacokinetic properties such as long serum half-life and low immunogenicity of MT201 and low internalisation of the antibody-bound target can positively impact the efficacy of an antibody in man. The prevalence of Ep-CAM in metastatic breast cancer and its prognostic relevance (Spizzo *et al*, 2002) make antibodies against this target very attractive for patients that are not eligible for trastuzumab treatment. According to a study by the Gastl group (Gastl *et al*, 2000; Spizzo *et al*, 2002), approximately 25% of patient samples analysed had high levels of Ep-CAM expression but were HER-2 negative. A total of 90% of all metastatic breast cancer patients had Ep-CAM-positive tumours, of which 42% showed high-level and 48% medium to low-level Ep-CAM expression. Not only tumours that express high levels of Ep-CAM molecules on their surface but also certain tumours that express low to intermediate levels may be susceptible to ADCC by MT201, as suggested by the data presented in this report (see Table 3). Treatment with MT201 for several months at high trough levels may provide ample time for MT201 to recruit immune effector cells for elimination of tumour cells with a broad spectrum of Ep-CAM expression levels.

## Figures and Tables

**Table 1:** Properties of human breast carcinoma cell lines

	**Primary tumour**	**Origin**	**Comment**
MT-3	Breast carcinoma	Mammary gland	Passaged in nude mice
SKBR3	Breast adenocarcinoma	Pleural effusion	HER-2 overamplification
MCF7	Breast adenocarcinoma	Pleural effusion	Oestrogen receptor positive
MaTu	Breast carcinoma	Mammary gland	Passaged in nude mice
ZR-7513	Breast ductal carcinoma	Ascites	Oestrogen receptor positive
BT20	Breast carcinoma	Mammary gland	HER-2 overamplification
MDA-MB-453	Breast carcinoma	Pleural effusion	FGF expression
MDA-MB-231	Breast carcinoma	Mammary gland	TGF*α* expression
BT474	Breast ductal carcinoma	Mammary gland	HER-2 overamplification

**Table 2:** Expression of Ep-CAM and HER-2 on human breast carcinoma cell lines

**Cell line**	**Ep-CAM expression^a^**	**HER-2 expression^a^**	**Ep-CAM : HER-2 ratio**
MT-3	671.2 (±123.1)	27.4 (±2.7)	24 : 1
ZR-751	298.2 (±98.2)	68.9 (±3.8)	4 : 1
MCF7	222.1 (±13.7)	25.2 (±1.6)	9 : 1
MDA-MB-453	180.3 (±30.7)	160.7 (±73.5)	1 : 1
BT20	139.5 (±27.0)	41.1 (±6.1)	3 : 1
SKBR3	125.5 (±31.6)	976.2 (±292.8)	1 : 8
MaTu	123.9 (±34.2)	16.7 (±0.7)	7 : 1
BT474	122.0 (±40.0)	690.6 (±247.0)	1 : 6
MDA-MB-231	1.7 (±0.6)	14.1 (±1.5)	1 : 8
KATO III	893.1 (±166.7)	23.4 (±4.0)	38 : 1

a Number of binding sites (× 10^3^).

Saturation titration analysis was carried out as described in Materials and Methods. KATO III cells served as reference cell line. The mean numbers of binding sites from three different experiments and standard deviations are shown.

**Table 3:** ADCC-mediated target cell lysis by MT201 and trastuzumab

**Cell line**	**No. of experiments**	**MT201^a^**	**Trastuzumab^a^**	**MT201 : trastuzumab**
MT-3	3	41.4 (±10.5)	31.9 (±6.7)	1.3 : 1
ZR-751	3	13.5 (±4.3)	14.5 (±4.3)	1 : 1.1
MCF7	3	23.5 (±8.3)	25.3 (±11.1)	1 : 1.1
MDA-MB-453	3	44.3 (±7.2)	64.1 (±5.9)	1 : 1.4
BT20	3	6.7 (±2.8)	7.6 (±0.7)	1 : 1.1
SKBR3	3	8.3 (±4.9)	33.2 (±15.1)	1 : 4
MaTu	3	25.8 (±2.4)	19.8 (±4.7)	1.3 : 1
BT474	3	10.8 (±5.2)	36.5 (±3.8)	1 : 3.4
MDA-MB-231	3	6.2 (±1.7)	17.0 (±4.4)	1 : 2.7
KATO III	7	39.9 (±14.1)	20.5 (±11.7)	2 : 1

ADCC (antibody-dependent cellular cytotoxicity) is expressed as specific maximal cell lysis in percent of all target cells as observed in dose–response analyses (see Figure 1). The assay used human PBMC effector cells at an E : T ratio of 20 : 1 in 4-h assay. KATO III cells served as reference cell line. Specific maximal cell lysis refers to the percentage of target cell cytotoxicity detected in the presence of antibody above background spontaneous lysis. The mean values from the indicated number of experiments and standard deviations are shown.

a Specific maximal cell lysis (%).

**Table 4:** ADCC-mediated target cell lysis by MT201 and trastuzumab

**Cell line**	**No. of experiments**	**MT201^a^**	**Trastuzumab^a^**	**MT201 : trastuzumab**
MT-3	4	47.7 (±28.1)	20.5 (±7.2)	2.3 : 1
ZR-751	3	200.3 (±35.1)	15.7 (±9.3)	12.8 : 1
MCF7	3	100.8 (±99.9)	11.3 (±3.4)	8.9 : 1
MDA-MB-453	3	92.1 (±71.1)	4.4 (±1.3)	20.9 : 1
BT20	3	311.5 (±47.7)	138 (±106.7)	2.3 : 1
SKBR3	3	461.4 (±238.3)	16.1 (±9.1)	28.7 : 1
MaTu	3	262.3 (±249.0)	112.7 (±86.6)	2.3 : 1
BT474	3	11.9 (±6.5)	8.3 (±3.2)	1.4 : 1
MDA-MB-231	3	N/A	65.9 (±36.5)	N/A
KATO III	13	84.6 (±57.7)	117.3 (±71.1)	1 : 1.4

Antibody concentrations that triggered half-maximal cell lysis by ADCC (see Figure 1) are shown. The assay used human PBMC effector cells at an E : T ratio of 20 : 1 in 4-h assay. Numbers give mean values from the indicated number of experiments and standard deviations. KATOIII cells served as reference cell line. N/A: not assessed.

a EC_50_ values in ng ml^−1^.

**Table 5:** CDC of MT201 and trastuzumab and expression of complement resistance factors

**Cell line**	**CDC by MT201^a^**	**CDC by trastuzumab**	**CD46^b^**	**CD55^b^**	**CD59^b^**
MT-3	30–50	−	++	++	+
ZR-751	−	−	+	++	++
MCF7	−	−	+	+++	++
MDA-MB-453	−	−	+++	+	+
BT20	−	−	+++	+++	++
SKBR3	−	−	++	+++	+++
MaTu	−	−	++	+++	+++
BT474	−	−	+	+	+
MDA-MB-231	−	−	+	+++	+++
*KATO III*	*80–100*	−	*++*	+	−

CDC and the expression of complement resistance factors CD46, CD55 and CD59 was determined on all cell lines as described in Materials and Methods. Antibody concentrations up to 50 *μ*g ml^−1^ were tested. Expression of CD46, CD55 and CD59 was determined by FACS analysis using commercial antibody reagents.

a Specific cell lysis (%).

b −, no staining; +, weak staining; ++ intermediate staining; +++ strong staining.
